# Selection of best-performing reference gene products for investigating transcriptional regulation across silvering in the European eel (*Anguilla anguilla*)

**DOI:** 10.1038/srep16966

**Published:** 2015-11-23

**Authors:** Silvia Franzellitti, Alisar Kiwan, Paola Valbonesi, Elena Fabbri

**Affiliations:** 1 University of Bologna, Department of Biological, Geological, and Environmental Sciences, via Selmi 3, 40100 Bologna Italy; 2 Interdepartment Centre for Environmental Science Research, via S. Alberto 163, 48123 Ravenna Italy

## Abstract

The focus of the present study was to set a methodological approach for evaluating molecular mechanisms underlying silvering transformation in the European eel, *Anguilla anguilla*. Silvering is a tightly controlled process during which eels undergo significant morphological, physiological and behavioral changes, pre-adapting for the oceanic spawning migration. Female eels showing different silver indexes were caught in different seasons in the Comacchio Lagoon (North Adriatic Sea, Italy). Isolated hepatocytes from these eels were selected as the experimental model given the relevant role of these cells in metabolic functions potentially altered during silvering. Expression profiles of 7 candidate reference transcripts were analyzed seeking the most viable and robust strategies for accurate qPCR data normalization during silvering. Stability analysis and further statistical validation identified transcripts encoding the ribosomal proteins L13 and ARP as the appropriate reference genes in studies on *A. anguilla* through silvering. The identified reference transcripts were further used to evaluate expression profiles of target transcripts encoding the thyroid hormone receptor β (*THR*β) and vitellogenin (*vtg*), known to be involved in silvering processes. To the best of our knowledge, this is the first study comparing *THR*β expression in European eels across silvering.

The European eel (*Anguilla anguilla*) is a catadromous teleost, which exhibits a complex life cycle with spawning in the ocean and growing up in continental waters. After spawning in the Sargasso Sea, within the North Atlantic gyre, eel larvae follow the Atlantic currents towards continental waters of Europe and North Africa, where they metamorphose into glass eels. After reaching coastal waters, estuaries, lagoons, rivers and lakes, they turn into yellow (sexually immature) eels and remain for several years in continental waters until they undergo a pre-pubertal secondary metamorphosis called silvering and transform into silver eels^1^. The silver eels migrate back to spawning grounds, where they eventually mate and die^2^,^3^.

The European eel stock has decreased by 95–99% compared to its levels in 1960–80^4^, so that the species is now classified as “critically endangered” by the International Union for Conservation of Nature (IUCN)^5^. The exact cause for this phenomenon is unknown, but several factors have been put forward to explain such a decline, including anthropogenic drivers as overfishing, pollution, habitat degradation, parasites and diseases, as well as environmental drivers such as climate and ocean changes^3^,^6^,^7^. The freshwater stages are likely to be more exposed to anthropogenic stressors, since they inhabit coastal and transitional environments heavily impacted by anthropogenic pollution; moreover, they effectively bioaccumulate environmental pollutants due to their peculiar ecology (benthic feeding) and physiology (high fat content)^8^. It has been hypothesized that the accumulation of contaminants during the feeding stage could impair the quality of spawners^3^,^9^,^10^, hence compromising success of transoceanic migrations, and contributing to the observed dramatic decline of larval stage recruitment^11^,^12^.

Silvering at its different stages, as defined by Durif *et al.*^13^, is a tightly controlled and complex process during which eels undergo significant morphological, physiological and behavioral changes, pre-adapting the animals for the oceanic migration. Therefore, understanding the molecular basis of silvering processes is a critical issue for environmental physiologists and conservation biologists, in order to unravel the potential impacts of environmental stressors on these processes, and safeguard the adaptive potential of eel stocks under heavy anthropogenic pressures.

The recent advances in molecular biology techniques are providing effective tools in the study of the regulatory pathways involved in the physiological adaptations to the environment. Transcriptomic-based technologies with their capacity for in-detail expression analysis of key gene features are pointing out crucial insights into molecular mechanisms at the basis of adaptative processes^14^,^15^. In particular, quantitative real time PCR (qPCR) is increasingly used in studies with environmentally relevant species, as changes in mRNA levels represent the earliest signals of physiological alterations that can potentially forecast changes at higher levels of the biological organisation^14^.

The application of qPCR assays in eel studies has emerged as a result of recent progress in eel genomics and transcriptomics due to the increasing amount of available sequences in public databases as well as to the application of high-throughput technologies, such as DNA microarrays or Next Generation RNA sequencing^16^,^17^,^18^, which have enabled the identification of gene products involved in key regulatory pathways.

qPCR technology is by far the most widely used method for the measurement of transcript abundance, representing to date the gold standard amongst molecular techniques employed in transcriptomic studies^19^. It is sensitive, specific and reproducible even with limited mRNA copy numbers^20^. Normalization is an essential component of a reliable qPCR assay because this process controls for sample-to-sample variations enabling comparisons of mRNA concentrations across different experimental conditions. The most commonly used method is normalization to the expression of an internal control transcript, also called reference gene product. Hypothetically, the ideal reference gene product is expressed at stable levels irrespective of tissue type, species, treatment, metabolism or sampling conditions. To date, no such ideal gene has been found and most likely does not exist. Several studies show that using single or inappropriate reference genes for normalization may dramatically bias the results of mRNA levels quantification^21^,^22^,^23^. Multiple sets of reference genes have been reported for human and mammalian studies in general, and experimental validation of reference gene stability is increasingly demanded as a pre-requisite for qPCR data publication (as an example, see Jacob *et al.*^21^). On the contrary, in studies with non-model organisms the lack of large set of genomic data has heavily limited these approaches; however, an increased awareness of the importance of systematic validation of reference gene selection is raised also by taking into account the potential confounding factors given by environmental pressures on animal transcriptional profiles evaluated under field conditions^10^.

Recently, Setiawan & Lokman^24^ investigated expression stability of 6 putative reference gene products in the New Zeland shortfinned eel (*Anguilla australis*) to infer silvering and the involvement of hormone stimulation in this event. The present study employed a set of 7 candidate gene products, which partially encompasses those assessed by Setiawan & Lokman^24^, to establish a viable and robust strategy for accurate normalization of qPCR data across silvering in the European eel. The identified reference transcripts have been further used to investigate differential expression of relevant gene products (namely thyroid hormone receptor and vitellogenin) between yellow and silver eels collected in the same area (Comacchio Lagoon, North Adriatic Sea, Italy) in different sampling periods.

## Results

### Amplification

A set of 7 candidate transcripts has been assessed (Table 1) in 4 groups of eels collected in different seasons and showing different Silver Index (SI) values (yellow eels collected in October 2013 and April 2014, henceforth addressed as YE13 and YE14, respectively; silver eels collected in October-November 2013 and November-December 2014, henceforth addressed as SE13 and SE14, respectively; Supplemental Figure S3). Amplification of each reference gene products in 32 samples (two replicates per sample) produced a 64-C_T_ (threshold cycle) values dataset. There were no samples with missing C_T_ or with inconsistency between replicates (C_T_ SD < 0.5). A single amplification product for each primer pair was confirmed by a single peak in the melting curve analysis and by electrophoresis analysis and sequencing of PCR products. After averaging replicates, descriptive statistics was obtained for each candidate reference gene product (Table 2). *EF1* showed the highest expression (C_T_ mean = 18.6), while *TBP* showed the lowest expression (C_T_ mean = 28.95). *L13* showed the lowest standard deviation (SD) while *CYTb* the highest.

### Analyses of candidate reference transcript stability

**Figure 1:**
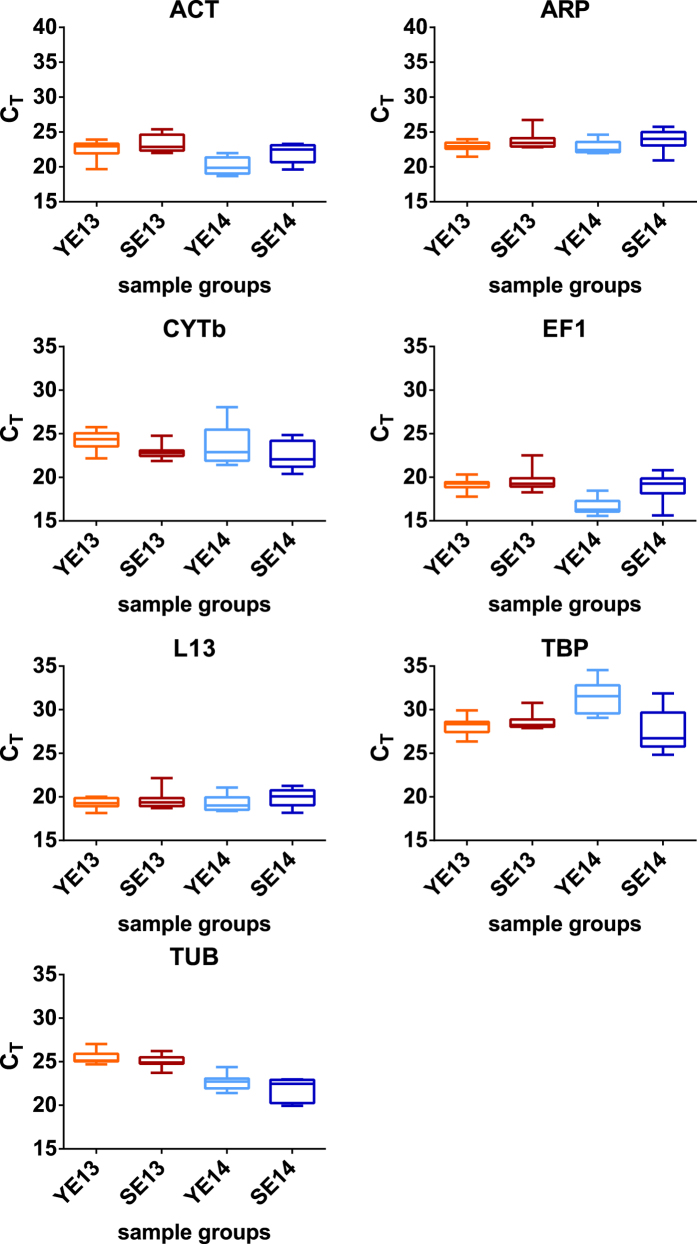
Intergroup transcript level variability of each candidate reference gene product in isolated hepatocytes from European eels. Data are based on threshold cycle (C_T_) values of candidate reference transcripts on isolated hepatocyte samples from eels at different silvering stages. Box-and-whisker plots represent median, upper and lower quartiles (n = 8).

**Figure 2:**
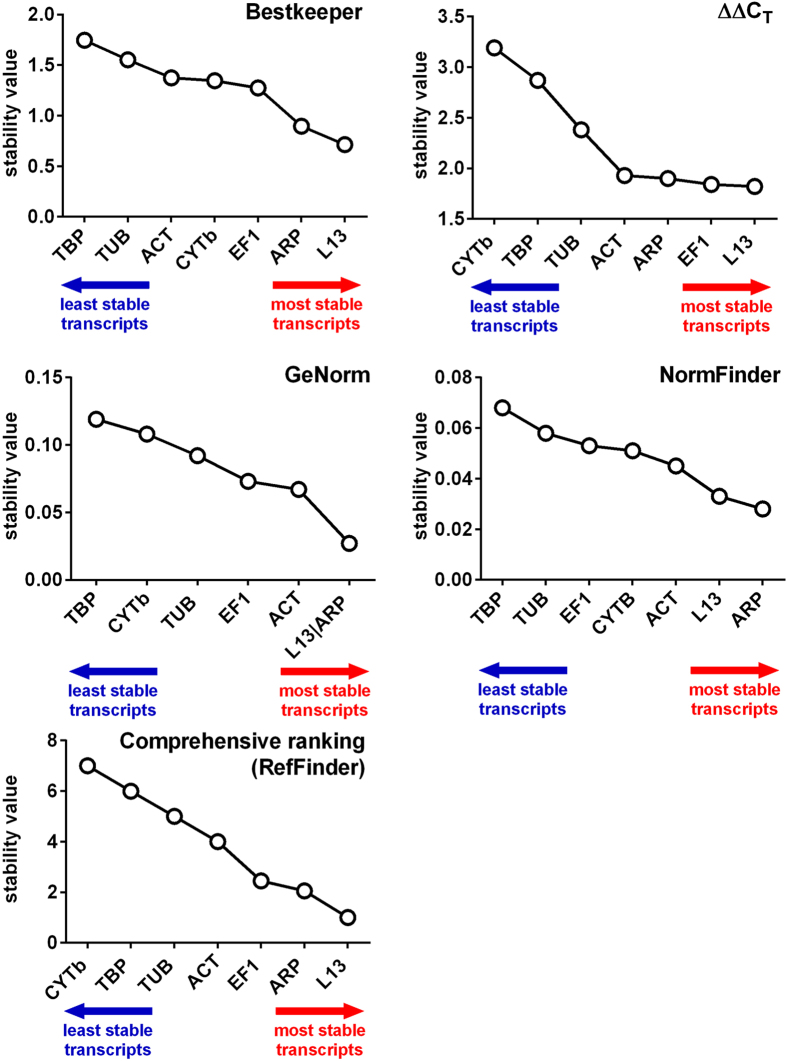
Stability rankings of the candidate reference gene products obtained using different computational methods.

Examination of overall C_T_ values showed variability among the different candidate reference gene products (Table 2), with the most stable and variable transcripts being *L13* and *TBP*, respectively. When considering intergroup variability, certain transcripts showed significant indications of being regulated across the different groups of eels (Fig. 1). In particular, *ACT*, *EF1*, and *TUB* showed higher mean C_T_ values in YE13 and SE13 samples with respect to YE14 and SE14 (Fig. 2). *TBP* showed higher mean C_T_ values in the YE14 sample with respect to all the other sample groups (Fig. 1). Again, *L13* showed the most stable expression levels across the different groups (Fig. 1).

Table 3 highlights different stability rankings of candidate reference gene products within different subsets of eel samples, and also a lack of agreement between the different algorithms employed. When stability analysis is performed using the whole set of data (Fig. 2), there is a general and broader agreement among the different programs used to rank the candidate reference transcripts according to their expression stability across the different eel silvering stages. *L13* was consistently the top-ranked or part of the top-ranked pair of transcripts, while *TBP* and *CYTb* resulted the worst performing gene products in our experimental conditions (Fig. 2). A comprehensive ranking calculated using the RefFinder web-based computational tool is also reported in Fig. 2.

**Figure 3:**
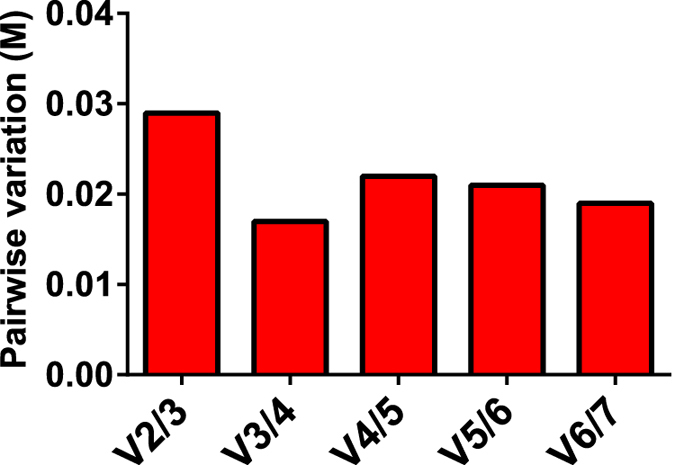
Determination of the optimal number of reference transcripts for normalization by geNorm analysis carried out on all eel samples. Average pairwise variations (Vn/n + 1) are calculated between the normalization factors NFn and NFn + 1, to indicate whether inclusion of extra reference transcripts adds to the stability of the normalization factor. Every bar represents the change in normalization accuracy when stepwise adding more internal reference transcripts according to the ranking in Fig. 2. Values < 0.15 are assumed to be sufficient.

Additionally, the pairwise variation of normalization factors derived from increasing numbers of gene products was calculated using geNorm, allowing estimation of the suitable number of reference transcripts needed for optimal normalization. As a pairwise analysis, 2 genes is the minimum, though additional normalization transcripts may reduce variation. However, this is neither guaranteed nor indeed always necessary. Values below 0.15 are generally accepted as sufficient^25^, thus as shown in Fig. 3 increasing the number of normalization genes from 2 to 3 or more is beneficial but unnecessary.

### Validation of selected reference gene products

Based on the stability analysis of candidate reference gene products, L13 was selected as the best performing single reference gene product and L13 and ARP were selected as the best performing combination of reference gene products (L13|ARP) to be used for normalization of real time PCR data in eel hepatocytes. Statistical validation analysis of these selected normalization approaches was performed using either the REST software or the Mann-Whitney U-test, and showed that expression levels of both L13 and the pair L13|ARP were not significantly different between the different eel silvering stages (Table 4), confirming their stable expression.

**Figure 4:**
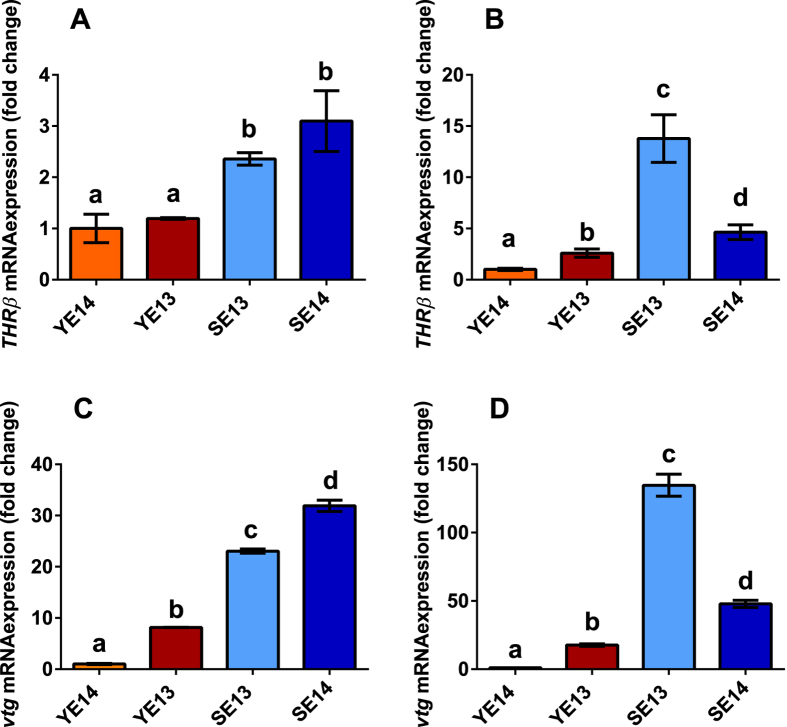
Normalized expression of the vitellogenin (*vtg*) and the thyroide hormone receptor beta (*THR*β) mRNAs in isolated hepatocytes from European eels at different silvering stages. Expression levels of *vtg* and *THR*β were normalized over the L13|ARP (A,C) or ACT (B,D) reference transcripts. Eel samples groups are ordered according to their average Silver Index (SI) (Supplementary Figure 3B). Values are expressed as the mean ± SEM (n = 8) of the relative variations (fold change) with respect to the YE14 group, which was selected as the reference sample group due to its lowest SI. Different letters indicate statistical differences (p < 0.05).

To validate the effectiveness of reference genes selected in the current study, the expressions of two transcripts, *THR*β and *vtg*, were analyzed in hepatocytes from European eels at the different silvering stages (Fig. 4). The data were normalized with the comparative delta-C_T_ method^26^. Both transcripts are expected to be up-regulated with increasing silvering stage in eels; indeed, they were found significantly up-regulated in both silver eel groups with respect to the yellow eel samples (Fig. 4A,C). Levels of *THR*β mRNA were not significantly different between groups of eels at the same silvering stage (YE13 *vs* YE14; SE13 *vs* SE14 Fig. 4A), whereas the *vtg* transcript showed significantly increased expression levels in the YE13 group with respect to the YE14 group, and in SE14 group with respect to SE13 samples (Fig. 4C). The Spearmann correlation test carried out using the SigmaStat software showed significant positive correlation between both L13|ARP-normalized mRNA expression profiles and the average Silver Index (SI) for each sampling group (*vtg*_*L13|ARP*_ *vs* SI: r = 0.93, p < 0.01; *THR*β_*L13|ARP*_ *vs* SI: r = 0.998, p < 0.001).

The L13|ARP normalization approach was further compared with normalization over the eel *ACT* expression, which was the normalization strategy previously employed both in *vtg* and *THR*β_*ACT*_ expression studies^27^,^28^,^29^,^30^. The Spearmann correlation test did not find significant correlations between both *ACT*-normalized mRNA expression profiles and the average SI for each sampling group (*vtg*_*ACT*_ *vs* SI: 0.44, p > 0.05; *THR*β_*ACT*_ *vs* SI: r = 0.38, p > 0.05).

## Discussion

In this study, isolated hepatocytes were used to set up a methodological approach for our ongoing investigations on transcriptional changes underlying metabolic adaptations of European eels during silvering and the possible influence of environmental stressors on such processes. This is in line with our previous studies addressing the physiological regulation of liver metabolism^31^, and the effects of environmental pollutants on such processes^32^,^33^. Besides our specific research interests, the qPCR normalization strategy proposed here may have a widespread application in the numerous experimental approaches employing transcriptional assays in fish hepatocytes (reviewed by Moon^34^).

Hepatocytes were isolated from female European eels caught in different seasons. Biometric parameters and calculated silver index (SI) showed that these eels were in different silvering stages, defined according to Durif *et al.*^13^; thus, expression profiles of 7 candidate reference transcripts were analyzed seeking the most viable and robust strategies for accurate qPCR data normalization during silvering.

As a general statement none of the available computational methods to check the stability of reference gene products is currently considered as the best one, and some problems may arise in certain experimental scenarios. Indeed, changes in reference gene suitability due to differences in study design, developmental stage, or physiological condition have been reported in many studies (locusts^35^, channel catfish^36^, sugarcane^23^, marine mussels^37^). All these potential confounding factors could be underestimated by the application of each of the computational methods, due to their mathematical constraints, so that external validation of the drawn conclusions seems advisable. Therefore, in this study we employed the approach proposed by Setiawan & Lokman^24^, which makes use of the REST 2009 software. REST 2009 is specifically designed for qPCR data analysis, so that it circumvents the difficulties of parametric statistical analyses on data based on proportions, such as those generated by qPCR^38^. Nevertheless, its conclusions were further corroborated by the independent Mann-Whitney tests, demonstrating that it is a suitable validating program.

Silvering involves systemic changes in physiology, morphology, and behavior, pre-adapting eels for the oceanic migration^39^,^40^,^41^,^42^. During this process body becomes silver, eyes and nostrils enlarge, muscles increase, gonads develop, and the atrophy of the alimentary tract takes place. These changes correlate with increasing gonadosomatic index and LH levels in the pituitary gland, leading to consider silvering as the onset of puberty rather a true metamorphosis^43^. A close relation between sexual maturation and metabolism has also been suggested, since the relevant endocrine modifications occurring during silvering are also accompanied by a significant metabolic shift between yellow and silver stages^3^,^44^,^45^. Indeed, in yellow eels energy stores rely mainly on glycogen^46^ while lipids become the main energy source in silver eels; moreover, silver eels do not eat during migration and energetic metabolism may be differently regulated with respect to yellow eels. It is likely that such metabolic adaptations could affect basal hepatocyte functions. Therefore, it is not surprising that transcripts encoding ribosomal proteins L13 and ARP, which showed stable and high expression levels^47^,^48^,^49^, resulted the best suited as reference gene products for normalization of qPCR data in hepatocytes from eels encompassing different silvering stages. This finding also agrees with a previous report on the seabass (*Dicentrarchus labrax*), in which *EF1 alpha* and *L13a* are addressed as suitable reference transcripts for an appropriate normalization approach during fish development, whereas *Fau* (40S ribosomal protein S30) and *L13a* are reported as the best choice when expression studies are performed using different tissues^47^. Setiawan & Lokman^24^ found that the combination of *18S* and *eef1* was the best suited to act as a reference gene products in the liver of the New Zeland shortfinned eel (*Anguilla australis*), whereas transcript levels for the 60 S ribosomal protein *L36* resulted the best reference transcript in ovary. In general, ribosomal RNAs (rRNAs) have been advocated for use as reference gene products in many experimental systems and their levels are thought to be less likely to vary under conditions that affect the expression of mRNAs, although other studies reported these transcripts being regulated under specific conditions^50^.

Validation analyses performed through REST2009 and the Mann-Whytney statistics showed that both the use of L13 and the combination L13|ARP represent a robust strategy for eel qPCR data normalization. However, the combination L13|ARP is preferred to obtain a more conservative estimate of mRNA expression changes and to accomplish with the MIQE guidelines, which require employing more than 1 endogenous control for qPCR data normalization^51^ as a more robust strategy for qPCR data normalization. As demonstrated by the geNorm analysis, using more than 2 reference gene products is not required under our experimental conditions.

Both the sampling season and the silvering stage seem to affect performance of other candidate reference transcripts, as clearly pointed out by *TUB* and *TBP*, that resulted regulated through at least one of those experimental conditions. This is further confirmed by the analysis of transcript stabilities within the sampling year or within yellow or silver eel stages, which gave different outcomes, and corroborate the hypothesis that constitutively expressed gene products underlying basal physiological functions (i.e. cell metabolism, protein synthesis) may be under endogenous as well as environmental regulation^52^.

The genetic background of eels may also be considered when analyzing samples collected in different seasons. Despite the fact that the European eel is commonly considered as a single, spatially homogeneous, randomly mating or panmictic population^53^, with mature adults likely sharing one single spawning area in the Sargasso Sea, weak though significant temporal genetic heterogeneity was observed between eels sampled in different periods, which may account also for differences in transcriptome-level responses^54^,^55^.

The suitability of the selected normalization strategy was assessed evaluating the expression profiles of target transcripts encoding the thyroid hormone receptor β (*THR*β) and vitellogenin (*vtg*), which are known to be involved in silvering processes. Vitellogenin is a female-specific yolk protein precursor, representing an energy reserve required for development of embryos in Teleosts as in other oviparous vertebrates. Transcription, translation and synthesis of this phospholipoglycoprotein takes place in female eel hepatocytes during vitellogenesis under the regulation of ovarian estrogens^56^. In males and in immature stages the *vtg* transcript is not expressed except in the presence of xeno-estrogens, so that its expression may serve as a biomarker of potential endocrine disruption by xenobiotics^57^. In Teleosts, thyroid hormones (THs) and their receptors (THRs) are involved in growth control, osmoregulation, and metamorphosis^58^,^59^. Up-regulation of *THR* expressions is known to anticipate metamorphosis in amphibian^60^, where elevation of both TH and estrogen levels is required for modulating *vtg* mRNA expression in hepatocytes competent for estrogen-dependent vitellogenin synthesis^61^,^62^. Significant correlations between levels of THs and vitellogenin were also observed in vitellogenic females of the common dentex (*Dentex dentex*), with maximum T3 and T4 concentrations being observed during final maturation^63^.

Recent investigations reported differential regulation of *vtg* and *THR*β transcript expression in female silver eels subjected to pituitary extract treatment (*vtg* up-regulation^27^,^29^), in male silver eels treated with steroids (*vtg* up-regulation^28^), and in eels naturally infected by macroparasites (*THR*β down-regulation^30^). To the best of our knowledge, this is the first study comparing *THR*β expression in different silvering stages of the European eel, whereas extensive investigations in many fish species, including the Japanese eel (*Anguilla japonica*) and the whitespotted conger eel (*Conger myriaster*), reported up-regulation of this transcript expression across larval development^64^,^65^. Accordingly, in the present study *THR*β was up-regulated in silver with respect to yellow female eels. As to *vtg*, a previous study from Blanchet-Letrouvé *et al.*^28^ showed that migrant silver female eels sampled in the Loire estuary exhibited significantly higher hepatic *vtg* mRNA levels compared to yellow females, yellow males and undifferentiated eels collected in the same area. Nevertheless, the authors failed to demonstrate a straightforward relationship between silver index and *vtg* mRNA expression across the analysed silvering stages^28^. It must be observed that the *actin* transcript has been employed as a reference gene for qPCR data normalization. However, from stability analysis performed in our experimental system this does not come out as a robust normalization strategy. Here we showed that when L13|ARP combination was used for qPCR data normalization, mRNA levels of *vtg* in silver eels were significantly higher compared to yellow stages, as reported by Blanchet-Letrouvé *et al.*^28^, and transcript profiles for both *vtg* and *THR*β were significantly correlated with the average SI. This correlation was lost when target mRNA expression levels were normalized over the *ACT* expression levels, thus corroborating the results of reference gene stability analysis reported in this study. Finally, mRNA expression data obtained in this study are in good agreement with data reported by Aroua *et al.*^43^, which showed moderate changes in thyrotrophic axis (both *TSH*β mRNA expression in pituitary gland and plasma levels of thyroid hormones) during eel silvering, while dramatic increases of vitellogenin levels in plasma.

## Conclusion

This study reports the use of an appropriate normalization strategy for qPCR investigations of silvering processes in European eels, showing that transcripts widely employed as reference gene products in previous investigations may be regulated across silvering stages or between different sampling periods. Local environmental selective pressures as well as peculiar genetic background of eels may also affect transcriptional processes. Transcripts encoding the ribosomal proteins L13 and ARP are suggested for an appropriate normalization approach in studies on *A. anguilla* through the silvering phases, although a critical evaluation of the stability analysis output along with external validation is advisable to overcome misleading results. The application of this normalization strategy led to a rigorous evaluation of the *vtg* and *THR*β transcript profiles, which are target transcripts relevant to sexual maturation processes. As such, the present work would provide an important methodological progress towards the establishment of hepatic *vtg* and *THR*β transcriptional analyses in eels and their potential use as markers of sexual maturation dynamics in this species of great economical and ecological relevance.

## Methods

### Eel sampling

Samplings of European eels (*Anguilla anguilla*, Linnaeus 1758) were performed in 4 different periods within the coastal lagoon of Comacchio (North Adriatic Sea, Italy). The Comacchio lagoon is a brackish coastal lagoon of about 100 km^2^ in surface area linked to the Adriatic Sea by two marine channels, and located about 20 km north of Ravenna, Italy (Supplementary Figure S1). The lagoon has historically been the site of extensive farming of European eels^2^ but has suffered from a rapid decline in the last 30 years. This decline has affected all European and Mediterranean eel populations and lowered the eel stocks to approximately 1% of their historical levels^4^,^66^. Silver eel capture was performed using the “lavoriero”, a V-shaped fixed traditional fishing weir^2^ which captures eels as they start moving from the lagoon towards the open sea. These captures took place in October-November 2013 (SE13) (mean water temperature 14 °C), and November-December 2014 (SE14) (mean water temperature 12 °C), since silver eels leave the Comacchio lagoon only during the winter season. Yellow eels were captured using nets in October 2013 (YE13) and April 2014 (YE14), when mean water temperature was about 15 °C. Eight specimen were captured for each sampling period. Median distributions of biometric parameters, including body length, body weight, eye index^67^, and pectoral fin length are reported in Supplementary Figure S2. Calculated condition factor^68^ and Silver Index^13^ (SI) values are reported in Supplementary Figure S3. SI consisted of five stages for female eels, represented by stages FI and FII (growth/trophic phase), stage FIII (pre-migrant stage characterized by the onset of gonad development), stage FIV (onset of the starvation and gonadotropin production) and stage FV (migrating stage characterized by digestive tract regression, pectoral fin elongation and higher gonadotropin levels). This classification allows a more precise and ecological description of the stages “yellow” and “silver” and could be used for the quantification of potential spawners from a catchment basin^13^. SE13 and SE14 eels were classified at the FIII stage (pre-migrating silver females) and at the FIV stage (migrating silver females), respectively (Supplementary Figure S3B). All eels in the YE14 group were classified as at the FII stage (yellow females, trophic phase), while 2 specimen of the YE13 eel group were classified as FIII (pre-migrant stage), and 6 as FII (Supplementary Figure S3B).

Eels were sacrificed by rapid decapitation. Hepatocytes were isolated by collagenase digestion of the perfused liver as described by Mommsen & Moon^69^, and preserved in RNAlater (Sigma Aldrich) at 4 °C for 24 h to enable proper sample stabilization, and then at −20 °C for long-term storage.

All experimental procedures were approved by the Ethical and Scientific Committee of Bologna University and were carried out in accordance with European legislation regarding the protection of animals used for experimental and other scientific purposes^70^.

### Primer selection

Candidate reference gene products as well as target transcripts and their functions are listed in Table 1. Primers were obtained from previous studies or were designed with Primer Express (Life Technologies) (Table 2), using nucleotide sequences retrieved from the GeneBank database (https://www.ncbi.nlm.nih.gov/genbank/) for *Anguilla anguilla* or from Next Generation RNA sequencing (454 FLX Titanium) of *A. anguilla* transcriptome (EeelBase database^16^). Primer pair specificities were checked both *in silico* and empirically by BLAST analysis and using melting profiles. BLAST analyses against *A. anguilla* nucleotide sequences in the GenBank indicated all primers were specific (data not shown), which was confirmed by melting profiles, electrophoresis, and sequencing of PCR products.

### RNA extraction and cDNA preparation

Total RNA was extracted from isolated eel hepatocytes using the ChargeSwitch total RNA cell kit (Life Technologies) according to the manufacturer’s protocol. DNAse I treatment was performed along with RNA extraction according to the manufacturers’ procedures (Life Technologies). RNA concentration and quality were verified using the Qubit RNA assay (Life Technologies) and electrophoresis using a 1.2% agarose gel under denaturing conditions. First strand cDNA for each sample was synthesized from 1 μg total RNA using the iScript supermix (Biorad Laboratories).

### qPCR assays

Real-time PCR (qPCR) reactions were performed in duplicate, in a final volume of 10 μL containing 5 μL iTaq Universal Master Mix with ROX (BioRad Laboratories), 2 μL diluted cDNA, and 0.2 μM (*L13, ARP, EF1, TUB, ACT, CYTb, vtg*) or 0.5 μM (*TBP, THR*β) specific primers (Table 2). A control lacking cDNA template was included in the real-time PCR analysis to determine the specificity of target cDNA amplification. Amplifications were performed in a StepOne real time PCR system apparatus (Life Technologies) using a standard “fast mode” thermal protocol. For each target mRNA, melting curves and gel pictures were analysed to verify the specificity of the amplified products and the absence of artifacts. The amplification efficiency of each primer pair was calculated using a dilution series of cDNA (Table 2).

### Selection of best-performing reference gene products

To calculate the transcript stability and identify the best performing candidate reference gene products, the algorithms BestKeeper^71^, geNorm^25^, and NormFinder^72^ were employed, along with the comparative delta-C_T_ method^73^. BestKeeper, geNorm, and NormFinder analyses were performed using Excel-based Macros, whereas Comparative delta-C_T_ analysis was carried out using the DataAssist software (Life Technologies). The RefFinder web-based tool was used to account for a comprehensive ranking calculated from the above-described algorithms (http://www.leonxie.com/referencegene.php?type=reference#).

BestKeeper calculates a geometric average for the entire dataset of C_T_, generating a hypothetical global normalization factor, the ‘bestkeeper’, taking the premise that as the number of transcripts measured increases, variations resulting from genuine biological changes in mRNA expression make progressively lower contributions to the overall trend than do global variations in cDNA concentration arising from sample preparation/handling. Thus, by making pairwise comparisons between expression data from individual transcripts and this ‘bestkeeper’, one can identify those with least variation, which best reflect overall cDNA (thus mRNA) levels. Reference gene products can be ordered from the most stable, exhibiting the lowest variation expressed as standard deviation (SD) or coefficient of variance (CV), to the least stable, exhibiting the highest variation.

geNorm considers that highly-similar patterns of variation observed in genes associated with unrelated cellular processes are more likely to reflect variation in cDNA levels than in mRNA expression. This technique utilizes pairwise comparisons to rank candidate gene products by summed individual variation; then it iteratively discards the most variant transcripts before repeating the analysis, thus identifying a pair of gene products with minimum respective variation, and a ranked list of transcripts of increasing variance. Transcript stability is expressed by a stability measure (M value) based on the geometric mean of all studied gene products and the pairwise variation. The lower is the geNorm M value the more stable is the gene.

Normfinder calculates individual variations for each gene relative to the dataset average, accounting for both intergroup (here comparing different groups of eel samples) and intragroup (here comparing individuals within each sampling group) variations, summing these to provide a gene-specific stability value for a given set of conditions. This inclusion of group-specific analysis identifies transcripts that show the greatest overall stability over the conditions presented, rather than those that merely exhibit well-matched variation. The lower is the NormFinder stability index the more stable is the gene.

RefFinder is a user-friendly web-based comprehensive tool developed for evaluating and screening for reference gene stability from extensive experimental dataset. It integrates the currently available major computational programs (geNorm, NormFinder, BestKeeper, and the comparative C_T_ method) to compare and rank the tested candidate reference genes. Based on the rankings from each program, it assigns an appropriate weight to an individual gene and calculated the geometric mean of their weights for the overall final ranking.

### Statistical validation and data analysis

Values of C_T_ and biometric parameters were compared between the different sampling groups using non-parametric one-way ANOVA (Kruskal-Wallis test) followed by the Mann-Whitney U-test (p < 0.05), after deviations from parametric ANOVA assumptions being verified (Normality: Shapiro-Wilk’s test; equal variance: F-test). These statistical analyses were performed using SigmaPlot ver. 12 (Systat Software).

The best reference gene product or combinations of gene products selected by the algorithms were statistically validated using the approach reported by Setiawan & Lokman^24^. The REST 2009 software^38^ was used to test for differences between the 4 groups of eel samples. This software is specifically designed to perform pairwise comparisons of qPCR data through randomization and bootstrapping techniques. The approach assumes that a stable reference gene product or a stable combination of reference transcripts would not show a stably expressed target transcript as being regulated. Hence, an artificial “dummy” transcript with a given uniform C_T_ value of 15 was set as the target gene product, so that by testing for differences between groups for the dummy transcript, validation for consistency of the selected normalization approaches was tested^24^. Furthermore, the Mann-Whytney U-tests (Sigma Plot) were also performed to directly test for differences in transcript abundances between eel groups. In both approaches, p < 0.05 was set as the threshold level of statistical significance. The power of the applied tests, as evaluated by the SigmaStat software, was always above 0.8 with p < 0.05.

Relative expression levels of *THR*β and *vtg* were calculated by a comparative delta-C_T_ method^26^ using the StepOne and Expression Suite software packages (Life Technologies) and applying the selected normalization approaches. Data were reported as relative expression (fold change) with respect to YE14, used as the reference group as it displayed the lowest SI, and statistically evaluated by the REST software to assess the significance of the differences in relative expression between each group and the reference group. Further comparisons between pair of treatments were performed using the Mann-Whitney U-test.

## Additional Information

**How to cite this article**: Franzellitti, S. *et al.* Selection of best-performing reference gene products for investigating transcriptional regulation across silvering in the European eel (*Anguilla anguilla*). *Sci. Rep.* **5**, 16966; doi: 10.1038/srep16966 (2015).

## Supplementary Material

Supplementary Information

## Figures and Tables

**Table 1:** Candidate reference gene products, target transcripts involved in eel silvering, and their functions.

Gene name	Gene abbreviation	GeneBank Accession Number	Function
β-actin	ACT	AY763793	Component of the cytoskeleton; mediates cell motility.
ribosomal protein L13	L13	eeel2_c1081*	Structural component of the large 60S ribosome subunit
TATA-box binding protein	TBP	eeel_c23519*	Transcription factor. TBP binds to the minor groove of DNA at the TATA box sequence, producing a large-scale deformation in DNA and initiating transcription.
Elongation factor-1a	EF1	EU407825	Catalysation of GTP-dependent binding of amynoacyl-total RNA to the ribosome; translational factor.
Cytochrome b	CYTb	AF006714	Component of the ubiquinol-cytochrome c reductase complex (complex III) in mitochondria.
Acidic ribosomal protein	ARP	AY763793	Member of ribosome proteins. It participates in the peptide elongation process by interacting with the GTPase domain in the ribosomes and the elongation translation factor eEF-2.
Tubulin	TUB	eeel_c9991*	Structural constituent of cytoskeleton; mediates cell motility.
Thyroid hormone receptor	THR β	AF302241	Hormone receptor
vitellogenin	vtg	AM076793	Female-specific yolk protein precursor

^*^Retrieved in the EeelBase database^16^: http://compgen.bio.unipd.it/eeelbase/.

**Table 2:** Selected candidate reference gene and target gene (vitellogenin, vtg; thyroid hormone receptor β, THRβ) primers and their parameters derived from qPCR analyses.

Gene	Primer sequences	Amplicon size(bp)	PCR efficiency(E%)	N	Mean C_T_	SD	CV(%)	Reference
Candidate reference gene products								
L13	5′-AAAGGAAGCGTATGGTGGTG-3′	180	95	32	19.51	0.93	4.77	Maes *et al.*^10^
	5′-CGGTCTTCTTCTTGCCGTAG-3′							
ARP	5′-GTGCCAGCTCAGAACACTG-3′	107	112	32	23.34	1.2	5.14	Weltzien *et al.*^74^
	5′-ACATCGCTCAAGACTTCAATGG-3′							
EF1	5′-GGCTGGTGGTGTAGGTGAGT-3′	183	94	32	18.58	1.62	8.72	Maes *et al.*^10^
	5′-TAAGCGCTGACTTCCTTGGT-3′							
TUB	5′-TCAGTCCCCACCTCTTCATAG-3′	133	98	32	23.74	1.82	7.67	This study
	5′-AGTTTGACCTGATGTACGCC-3′							
ACT	5′-CAGCCTTCCTTCCTGGGT-3′	226	100	32	21.99	1.72	7.82	Aroua *et al.*^75^
	5′-AGTATTTGCGCTCGGGTG-3′							
TBP	5′-TGGTATCACGGCACATTGTT-3′	180	98	32	28.95	2.26	7.80	Maes *et al.*^10^
	5′-ATGCGTAGATGTCCCTCCAG-3′							
CYTb	5′-CACAAATCCTTACAGGACTATTCCTAG-3′	200	100	32	23.33	1.65	7.07	Aroua *et al.*^75^
	5′-GTAAAGGTATGAGCCGTAGTAAAG-3′							
Target gene products								
THRβ	5′-ATTATCACCCCAGCCATCAC-3′	123	99	32	24.64	1.10	4.46	This study
	5′-CAGCGACATGATCTCCATACAG-3′							
vtg	5′-CCTACCACCAGCTTACCTTATG-3′	220	93.5	32	30.18	2.54	8.42	Blanchet-Letrouvè *et al.*^28^
	5′-CGCTGGGAGTGCGGAA-3′							

Gene abbreviations are as reported in Table 1. N: total number of samples; mean C_T_: mean C_T_ values; SD: standard deviations of the C_T_ values; CV(%): coefficients of variation of the C_T_ values.

**Table 3:** Stability rankings of the candidate reference gene products obtained with different computational methods.

Ranking	Comprehensive ranking (RefFinder)	Comparative delta-C_T_	BestKeeper	NormFinder	geNorm
2013 sampling season (YE13 AND SE13)					
1	L13 (1.19)	L13 (0.66)	TUB (0.54)	TBP (0.008)	L13|EF1 (0.02)
2	EF1 (2.06)	EF1 (0.67)	L13 (0.57)	L13 (0.012)	
3	TBP (3.13)	TBP (0.7)	EF1 (0.63)	EF1 (0.015)	ARP (0.023)
4	TUB (3.5)	ARP (0.79)	TBP (0.65)	ARP (0.021)	TBP (0.03)
5	ARP (3.94)	TUB (0.94)	ARP (0.70)	TUB (0.024)	ACT (0.038)
6	ACT (5.73)	ACT (0.98)	ACT (0.92)	ACT (0.026)	TUB (0.046)
7	CYTB (7)	CYTB (1.15)	CYTB (1.022)	CYTb (0.039)	CYTb (0.053)
2014 sampling season (YE14 AND SE14)					
1	L13 (1)	L13 (1.62)	L13 (0.86)	L13 (0.023)	L13|ARP (0.032)
2	ARP (1.86)	ARP (1.66)	TUB (0.91)	ARP (0.027)	
3	ACT (3.22)	ACT (1.87)	ARP (1.09)	TUB (0.048)	ACT (0.054)
4	TUB (3.76)	EF1 (1.94)	ACT (1.45)	ACT (0.054)	EF1 (0.066)
5	EF1 (4.47)	TUB (2.14)	EF1 (1.51)	CYTb (0.065)	TUB (0.088)
6	CYTb (6)	CYTB (2.67)	CYTB (1.63)	EF1 (0.075)	CYTb (0.116)
7	TBP (7)	TBP (3)	TBP (2.56)	TBP (0.098)	TBP (0.134)
Yellow stage (YE13 AND YE14)					
1	ARP (1.68)	ARP (1.47)	L13 (0.57)	L13 (0.029)	EF1|TUB (0.048)
2	L13 (2.11)	L13 (1.5)	ARP (0.66)	ARP (0.025)	
3	EF1 (2.28)	EF1 (1.58)	EF1 (1.35)	CYTb (0.038)	ACT (0.054)
4	TUB (3.34)	ACT (1.73)	CYTB (1.40)	TUB (0.049)	ARP (0.076)
5	ACT (4.12)	TUB (1.75)	TUB (1.44)	ACT (0.054)	L13 (0.078)
6	CYTB (5.42)	CYTB (2.31)	ACT (1.58)	EF1 (0.058)	CYTb (0.098)
7	TBP (7)	TBP (2.94)	TBP (1.88)	TBP (0.088)	TBP (0.116)
Silver stage (SE13 AND SE14)					
1	L13 (1.73)	EF1 (1.22)	L13 (0.80)	ACT (0.019)	L13|ARP (0.028)
2	EF1 (2.06)	ACT (1.26)	CYTB (0.91)	EF1 (0.020)	
3	ACT (2.38)	L13 (1.29)	EF1 (0.92)	TBP (0.022)	EF1 (0.051)
4	ARP (2.99)	ARP (1.3)	ACT (0.99)	CYTb (0.027)	ACT (0.059)
5	CYTB (3.98)	CYTB (1.38)	ARP (1.0)	ARP (0.031)	CYTb (0.069)
6	TBP (6)	TBP (1.73)	TBP (1.39)	L13 (0.035)	TBP (0.074)
7	TUB (7)	TUB (2.16)	TUB (1.62)	TUB (0.062)	TUB (0.092)

Stability values obtained by each method are shown in parenthesis. The gene products are ranked from the most stable (1) to the least stable (7).

**Table 4:** Pairwise statistical analysis of the inter-group stability of selected reference gene products.

Group comparisons	L13		L13|ARP	
	*p* based on REST 2009	*p* based on Mann-Whitney	*p* based on REST 2009	*p* based on Mann-Whitney
YE13 vs SE13	0.495	0.422	0.495	0.244
YE13 vs YE14	0.991	0.990	0.868	0.880
YE13 vs SE14	0.162	0.159	0.136	0.142
SE13 vs SE14	0.646	0.486	0.788	0.772
YE14 vs SE13	0.492	0.477	0.237	0.250
YE14 vs SE14	0.200	0.219	0.144	0.151
