# Supplementary material for: Selection of best-performing reference gene products for investigating transcriptional regulation across silvering in the European eel (Anguilla anguilla)
Source: Sci Rep. 2015 Nov 23;5:16966. doi: 10.1038/srep16966 (PMC4655329; doi:10.1038/srep16966)
Supplement: Supplementary Information [file srep16966-s1.pdf]

SUPPLEMENTARY INFORMATION TO

Selection of best-performing reference gene products for investigating transcriptional regulation across silvering in the European eel (*Anguilla anguilla*)

Silvia Franzellitti*, Alisar Kiwan, Paola Valbonesi, Elena Fabbri

*corresponding author

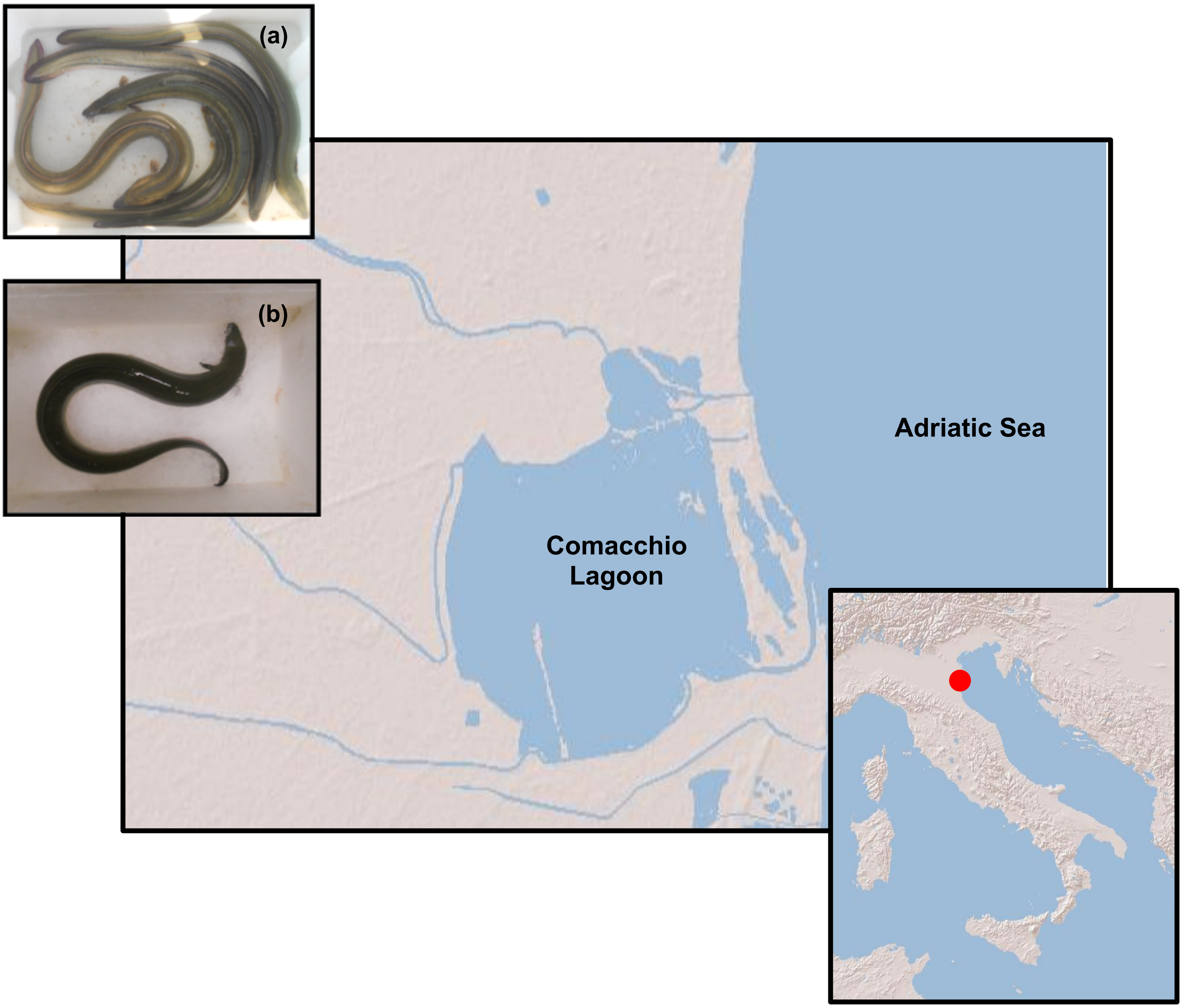

Figure S1. The sampling location (Comacchio Lagoon) in the North Adriatic Sea and examples of yellow (a) and silver (b) European eels collected in this study. Maps were generated and edited using the QGIS software ver 2.8. Eel pictures are from A. Kiwan.

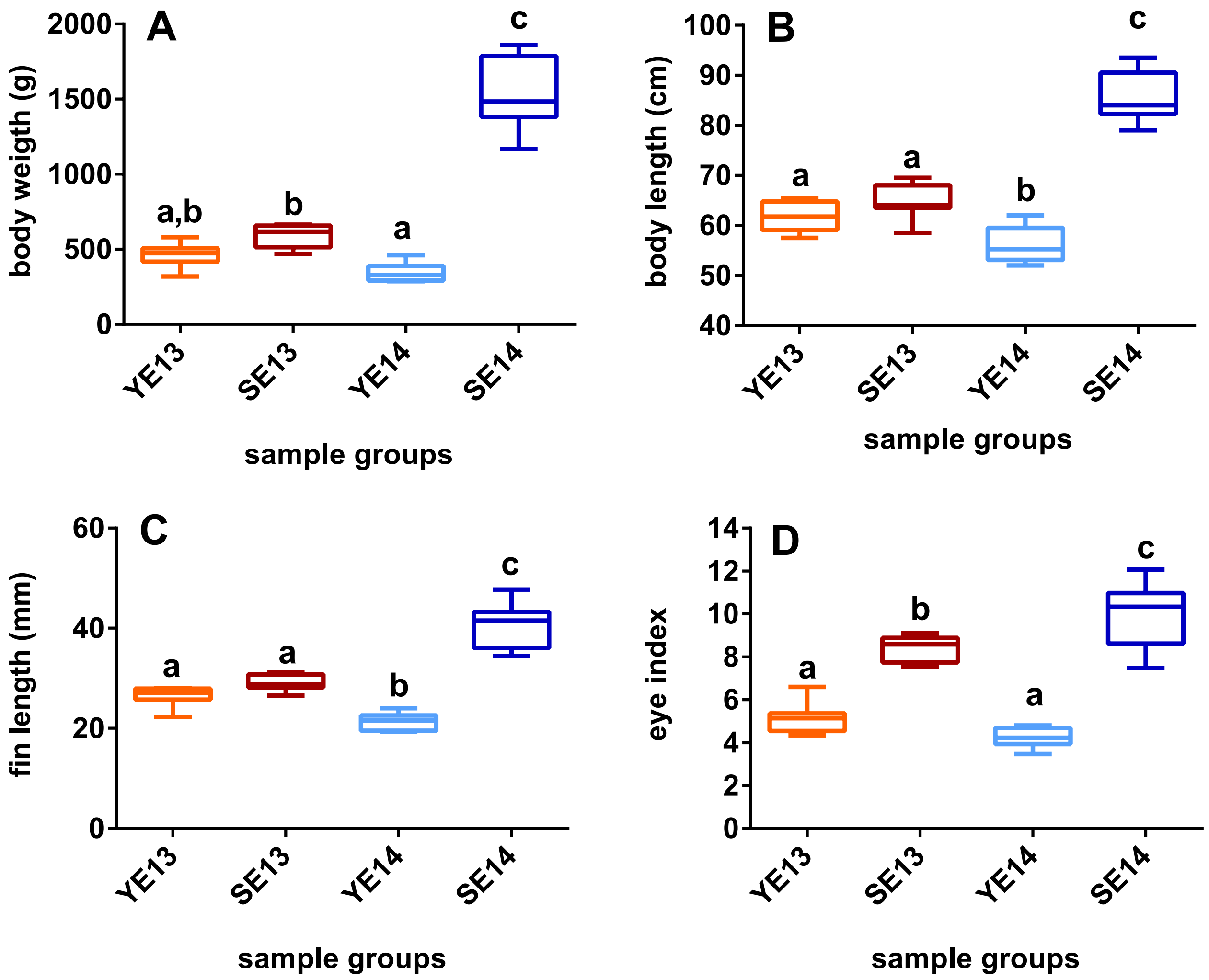

Supplementary Figure S2. Intergroup variability of biometric parameters assessed in European eels collected in the Comacchio Lagoon. Box-and-whisker plots represent median, upper and lower quartiles for body weight (A), body length (B), pectoral fin length (C), eye index (D). The Eye Index was calculated according to Pankhurst[67], using eye horizontal and vertical diameters, and total body length. Different letters indicate statistical differences ($p < 0.05$; $n = 8$).

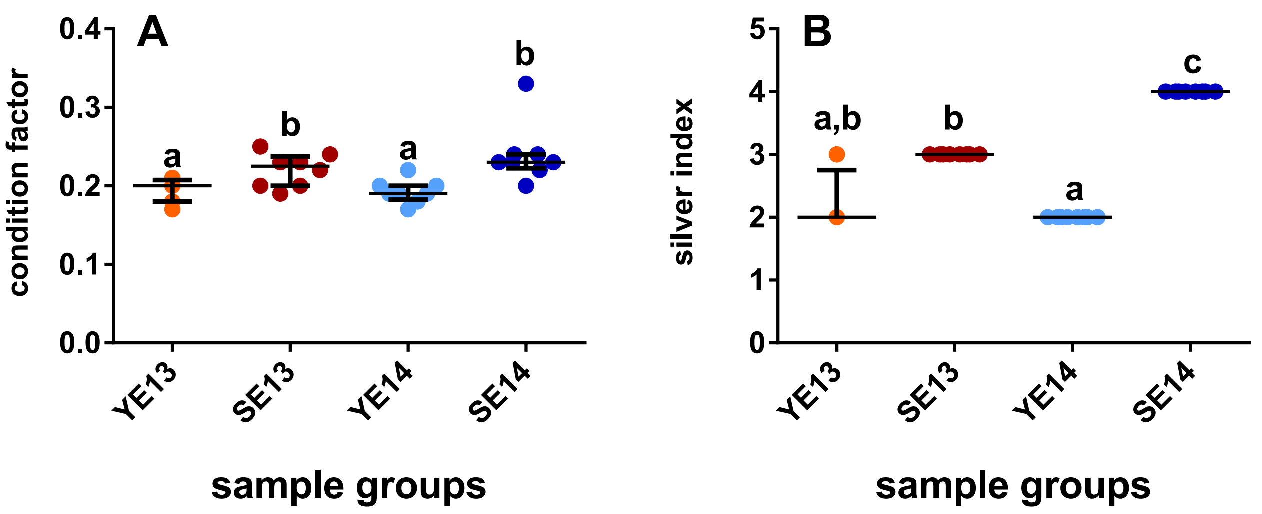

Supplementary Figure S3. Intergroup variability of calculated conditions factor (A) and silver index (B) values assessed in European eels collected in the Comacchio Lagoon. The condition factor was calculated according to Fulton[68] using total body weight and total body length. The silver index was calculated according to Durif *et al.*[13] taking into account total body length and weight, length of pectoral fin, mean eye diameter based on vertical and horizontal eye diameters. Horizontal lines indicate median values; error bars represent upper and lower quartiles; dots represent calculated values for the single eels within each group. Different letters indicate statistical differences ($p < 0.05$; $n = 8$).
